# Exogenous and endogenous factors affecting the social impact of cultural projects: the case of Barcelona ecosystem

**DOI:** 10.1186/s40410-023-00196-3

**Published:** 2023-03-15

**Authors:** Lluís Bonet, Giada Calvano, Pablo Fernández Compañ

**Affiliations:** University of Barcelona, Barcelona, Spain

**Keywords:** Cultural democracy, Social impact, Barcelona, Endogenous and exogenous factors, Cultural projects

## Abstract

Cultural projects, particularly when aiming at social impact, are usually the result of the context, values, and social fabric. Some endogenous factors, such as governance models and availability of resources, mainly committed human resources, could explain their particular development as well, despite being widely recurrent. Cultural policies are also determined by dominant social values and usually respond to the territorial context. But to what extent are these also enabling factors for the development of cultural projects with specific social goals? The present article intends to study the emergence of cultural projects with a clear intentionality of social impact in the local context of Barcelona. Starting from the analysis of 14 cases, the article evaluates the exogenous and endogenous factors and typologies of programmes implemented in order to understand the non-linear complexity of social impact generation in the case of cultural projects.

## Introduction

The financial crisis starting in 2008 fostered the channelling of cultural projects that explicitly seek a social impact in terms of health and well-being, enhanced citizen participation or urban regeneration, along with the emergence of public and private cultural policies that support these. Meanwhile, it boosted the precarity—and even the phasing out—of many cultural projects, especially in the South and East of Europe, being the areas that suffer most from reductions in public budget dedicated to culture (Bonet, Donato 2011, Čopič et al. 2013, Rubio Arostegui, Rius-Ulldemolins 2020). The COVID-19 crisis further intensified this trend, in particular regarding the health and wellbeing dimension (Stantcheva 2022; Paremoer et al. 2021, Martinez-Bravo, Sanz 2022). An increasingly impoverished and vulnerable local context, with direct effects on its cultural actors, leads to greater awareness of the role that cultural practices may have in terms of citizen welfare, autonomy, and engagement.

The dominant paradigm of cultural policies since the 1960s—cultural democratisation—has long been in crisis face to the inability of projects and institutions to break the social glass ceiling which limits the enjoyment of subsidised cultural practices mainly to upper- and middle-class collectives, with the corresponding cultural capital (Urfalino 1996; Evrard 1997; Bellavance 2000, Hadley, Belfiore 2018). At the same time, a more eclectic and postmodern view on aesthetic values and the criteria that allow evaluating quality leads to a progressive loss of glamour and social legitimacy (Marx 2022), which had allowed to have adequate resources for creating and later developing most of the existing cultural venues and programmes (Bonet, Négrier 2018a). As a result of this, and boosted by the wider economic and sanitary crises, the recuperation of the cultural democracy paradigm, which gives emphasis on cultural rights and bottom-up expressions (Négrier, Teillet 2022), such as participatory and community art (Rancière 2008; Matarasso 2019), has gained relevance. Nevertheless, this implies a need to enlarge the boundaries of the culture-based policies debate, questioning ‘what the arts can do for society’ (Matarasso 1997).

If since the turn of the century the legitimising instrument was essentially the economic impact, related to the new paradigm of creative economy, during the second decade of the twenty-first century it will also be the social impact (Belfiore 2006). Both could be seen as a complementary source of impact, since investments in culture do not only aim for economic benefits (employability, entrepreneurship, return on investment, etc.), but its externalities generate social benefits as well (Throsby 2010). Nevertheless, some scholars highlight the limits of economic approaches in evaluating the complexity of other intangible social dimensions of cultural activities (Belfiore, Bennett 2007).

Given the above considerations, what do cultural experiences bring about in terms of social impact? The social impact of culture could be understood from a multi-dimensional perspective, affecting the individual, collective and societal dimensions (Throsby 2003). From an individual viewpoint, cultural practice has the capacity to influence several impact areas, ranging from cognitive development, attitudinal changes, perceptions and motivations, health improvements and wellbeing (Azevedo 2016). Regarding the capacity for collective change, community impacts cover the promotion of social contact, interaction and social inclusion, enhancement of the sense of community identity, development of the ability to cooperate on a social issue, to engage citizens and to build social capital (Charlton et al. 2013; Secker et al. 2007; Williams 1996). Finally, culture could be a driver for social change and development (Tubadji, Osoba, Nijkamp 2015) through its wider impacts on territory—that cover not only elements such as physical and immaterial fabric and urban requalification, but also its economic and political aspects (e.g., the capacity of cultural activities to increase employability, entrepreneurship, city attractiveness, etc.) (Elden 2021).

The discourse around the social impacts of culture cannot avoid tackling the wider role that culture has in sustainable development: ‘adding the societal impact dimension of performance might then contribute to the advocacy of culture as fourth pillar of sustainable development, while deepening the understanding of value assessment in the creative industries and its multi-layered dimensions’ (Sabatini 2019: 35).

The greatest challenge consists of determining the impact dimensions to evaluate, as well as the evaluation criteria, and having available evaluation models which allow measuring the real achievement of those impacts (Hadida 2015). Few empirical research has focused on how specific cultural projects generate societal value, their sustainability and evaluation (Vermeulen, Maas 2021). In many cases, this is due to a lack of robust evidence, for instance in the case of culture-led regeneration processes (Evans 2005). Indeed, most studies try to deal with the intangible and invisible nature of these impacts by adopting qualitative measures, but qualitative appraisals also present limitations to evaluation and complex methodological faults (Azevedo 2016).

Nevertheless, some experiments to capture this complexity have been carried out. For instance, Gallou and Fouseki (2019) propose an adaptation of the social impact assessment (SIA) principles to evaluate the contribution of cultural heritage to social sustainability, supporting both a people-centred and socially responsible approach. Or Galloway (2009) considers theory-based evaluation (TBE) approaches, that allow the evaluation of public policy interventions at different stages of implementation, for understanding how and why arts engagement can result in social change.

Another possible strategy consists in basing the approach on the Logic Model of inputs- activities-outputs-outcomes-impacts (Anderson et al. 2011, Savaya, Waysman 2005), which results useful to map the elements in play in social transformation processes. However, this model does not allow to seize the complexity of impact generation, which cannot be reduced to a linear sequence, thus requiring an understanding of the different relationships and exchanges in action, for instance through theories of change (Vegel 2012).

Is it possible to build a causal relationship between citizen participation in cultural projects and direct effects at the individual, community, and territorial levels? Most social impact is produced in the mid and long term, fact that generates a number of issues: on the one hand, the required longitudinal approach is connected with the difficulty to isolate the direct impact of a project from other external factors; on the other hand, the intangible dimension of social externalities hinders the measurability of impacts.

A further aspect to consider is the relationship between the development of projects and supporting policies, by analysing the emergence of new social values and how these translate into policies. An increasingly widespread interpretation of urban and socioeconomic reality and the capacity of some cultural experiences to generate social transformation led to both the birth of new cultural projects and the rise of related new lines of public and private support. In fact, a two-way process has emerged: on the one hand, promoters of cultural projects with an explicit social orientation urge the generic programme schemes supporting culture to take into account this specific orientation. And, on the other hand, to the extent that a social re-legitimation of the cultural action is required, specific strategic—governmental and philanthropic—policies emerge, giving support to cultural projects with social impact.

As the social, cultural, and economic contexts get blurred (a fact that explains their birth and wealth), does their capacity to expand and replicate also weaken? Is it possible to generalise the emergence of projects and the proliferation of strategies of public and private support with social orientation to other temporal and territorial contexts? Which other enablers, beyond the effects of the crisis, may explain an increasingly social orientation of cultural initiatives and related public and philanthropic support policies? How to measure their impact? Some empirical research tries to answer some of these questions (Boix Domènech, De Miguel Molina, Rausell Köster 2021). One way of doing it is focusing on the analysis of how exogenous and endogenous factors affecting cultural projects explain the relationships between projects and policies (Wilson, Gross, Bull 2017). Among the exogenous factors affecting cultural projects, the socioeconomic context, the social values on the dynamics of the cultural ecosystem, and the existence of explicit public and private policies have been highlighted (Montalto et al. 2019, Tubadji, Osoba, Nijkamp 2015). Regarding endogenous conditions, cultural management literature identifies the governance model and organisational culture, the availability of committed human resources, and the availability of material and financial resources (UNESCO 2013, Bucci, Sacco, Segre 2014, Anttonen et al. 2016).

The authors of this article argue that endogenous factors (governance model and organisational culture, and human, symbolic, material and economic resources) are intertwined with exogenous conditions (development of public and philanthropic support policies, the urban and socioeconomic context, and the social values and dynamics of the cultural ecosystem). The differentiation between exogenous and endogenous factors lies in the cultural project’s agency capacity, since the project is the core object of the present article.

Starting from the analysis of a set of cultural projects aimed at social transformation in the Barcelona ecosystem, the article tries to demonstrate that endogenous and exogenous factors affect the cultural strategies of the projects, the choices related to the selection of the typology of cultural programmes and the beneficiaries’ target groups. In turn, these latter are closely linked to the desired social outcomes that organisations’ mission and values aim to achieve.

## Methodology

The empirical and conceptual approach used in this paper benefits from the results of the MESOC project (H2020), focused on the assessment instruments of measuring the social impact of culture in three main dimensions: health and wellbeing, participation and civic empowerment, and urban regeneration. The analysis has focused on the specific context of the Barcelona metropolis, and the experiences of 14 selected cultural projects looking explicitly for social impact.

The Barcelona ecosystem is particularly pertinent as a case study since the municipal government, during the analysed period, put cultural rights and social impact at the heart of the political debate and the grassroots practice (Barcelona City Council 2022). Additionally, the pre-existence of an engaged civil society and of shared values and fabric represent a favourable cultural hummus which is progressively activated as a reaction to the effects of the 2008 economic crisis, both at the social and political as well as the cultural level (Blanco 2018). This dialectic phenomenon is the result of a top-down and bottom-up synergic dynamic that exemplifies current literature debate (Baldo, Demartini 2012) that is relevant for understanding how emerging cultural initiatives connect with explicit public policies.

Given the above, the methodology of case studies’ analysis has been considered the most adequate for the scope of this paper, since this method has been found especially valuable in the study of complex and heterogeneous systems in social science research (Morgan 2012).

In order to select the projects included in the empirical analysis of Barcelona, given the non-existence of official databases on this typology of projects, officers from the City Council, private foundations supporting cultural projects with social impact, and the Art i Barri Network^1^ have been consulted. The research resulted in a first list covering 50 projects. From this list, 14 projects were selected after filtering the sample according to the following criteria:

- Explicit inclusion of social impacts in the projects’ objectives;
- The social impact should cover minimum one of the three main dimensions of the New European Agenda of Culture classification (European Commission 2018);
- Cultural projects should be carried out within the Barcelona metropolitan geographical area, and take place between 2015 and 2020^2^;
- Diversity and complementarity of cultural expressions (music, literature, audio-visual, gastronomy or urban art, among others), and type of programmes (co-creation, volunteering, capacity building, historical or autobiographical memory, non-formal art education, etc.).

A short description of the selected projects is provided in Table 1.

**Table 1:** Case studies

Project number	Name of the project	Name of the organisation	Short description
1	Apropa Cultura	Orchestra and Auditorium of Barcelona Consortium	Apropa Cultura is a programme oriented to fostering and diversifying access to cultural venues, making their programming accessible and comprehensible for everyone, thanks to a relevant effort of mediation and communication. It addresses disadvantaged and vulnerable social collectives, who may face entry barriers (people with intellectual disability, people with physical impairment, people with mental issues, women, the elderly, addicts, or migrants). The aim is to democratise culture, with all its transforming power, and showing another panorama to the citizens who could not usually enjoy access to culture. These activities pretend to help these collectives in their path towards inclusion and equality
2	En Palabras [Relatos migrantes]	Connectacts Cooperative	En Palabras [Relatos migrantes] is a collective project of literary creation and editing, that reunites refugees, migrants, and writers from all around Latin America who are residents in Barcelona. Its aim is to develop a project of communitarian documentation and of construction of memory around the situations of violence and rights violation that oblige people from the Latin America region to abandon their native lands, and around the difficulties they face during the travel and when they arrive at the host destination
3	Xamfrà	L'ARC Música Foundation	Created in 2004, Xamfrà is a socio-educational centre that uses the arts to guarantee the right of access to cultural and artistic practice for all citizens, according to the objectives of the L’ARC Música Foundation. These objectives revolve around principles of equality/equity, recognition of diversity, positive interaction, diverse participation, and feelings of belonging. Since 2014, Xamfrà has been dedicated to the promotion of social inclusion through music, dance and performing arts
4	Womart	Rebobinart Association	Womart is a project aimed at claiming the need to recognise feminine talent in visual arts, in general, and in street art (or art in public space), in particular. The lack of gender equality in the institutional circuits of visual arts reveals that there still exists a glass ceiling that prevents achieving real equality in terms of recognition of women professional artists. Womart was born in response to this reality, with the objective to make visible and acknowledge the artistic talent of women creators and to foster gender equality in all visual arts’ disciplines
5	Explainers	La Caixa Foundation	Explainers is a project carried out at CosmoCaixa, a science museum that is part of La Caixa Bank Foundation. The programme was inspired by the initiative of Science Museums in the USA, which since 1969 is the flagship programme of the Exploratorium in San Francisco. Explainers are students between 15 and 18 years old of different schools of Barcelona, that accompany the museum visitors with didactic and creative explanations. The programme offers the opportunity to discover science from a different perspective, while practising competences and skills such as teamwork, communication, adaptation to change, commitment to a project, creativity, autonomy, etc
6	Primer Cinema	La Caixa Foundation	Primer Cinema is an audiovisual educational pilot project. It was born from educational experiences in the audiovisual area and especially after the contest and educational project “Participa Méliès”. Primer Cinema proposes to school professors to take part with their students in an educational project focused on the audiovisual medium, and the introduction to this language (mixing mute cinema and special effects), to promote methodologies that reinforce transversality among arts and knowledge and strengthen collective creation dynamics, while working on questions of digital citizenship, in particular related to dissemination tasks
7	Art i Part	ICUB—Culture Institute of Barcelona	Art i Part communitarian artistic creation programme in the neighbourhoods was an initiative of the City of Barcelona, managed through its Cultural Institute (ICUB). It invited neighbours of the city to take part in a communitarian artistic proposal where they took part as creators. In 2018, Art i Part developed the first edition in five neighbourhoods of the city, whilst in 2019 it focused on other three neighbourhoods: Besòs, Gòtic and Poble Sec
8	Basket Beat	Basket Beat Association	Basket Beat is an entity, a project and a methodology to accompany people, especially the less advantaged ones, in their personal growth through music creation and learning with basketballs. The main goal is to foster the meeting among people and critical thinking, as well as accompanying the institutions and venues they work with. They organise their work around three areas: 1) socio-educational workshops in schools, institutions, prison facilities, public venues, etc. 2) research and publications, as well as training sessions for students and professionals on the social use of arts, on their methodology and on the rethinking of the professional role. 3) the “basketball orchestra”, which provides career opportunities for young people and is used also as a team-building tool.. In this context, FAACCC (Community Arts Festival) was born, following and giving recognition to several cultural proposals with the aim to become a “radical” meeting among neighbours, professionals, organisations, academics, institutions and students around cultural participation and community arts
9	BiblioLab Cuines del Món	Fondo library	Bibliolab Cuines del Món had 2 different projects: Cuinem Santa Coloma (2018–2020) and Cuina Oberta (2020–2021). Cuinem Santa Coloma was born from the introduction of a kitchen in the Biblioteca del Fondo (Fondo library), generating a number of activities that get citizens closer to the venue of the library. This will to get all publics closer to the venue accounts the diversity, in terms of culture, ages, geographical origins, of the population of the Fondo neighbourhood. Tales and stories from all over the world are used to work the bond with children. Cuina Oberta proposes the creation and dynamisation of a user-friendly and inclusive space to reconnect with the gastronomy and local food. The programme aimed at promoting values of health, care, sustainability and culture, through the collaborative design of activities and the involvement of different citizens and collectives
10	ISC2: BiblioLab on social innovation and citizen science in territorial transformation in the Vallès Occidental	3 public libraries of the Valles Occidental: Central Library in Cerdanyola del Vallès, Vapor Badia Library in Sabadell, Volpelleres Miquel Batllori Library in Sant Cugat del Vallès	The project aims at maintaining the active role of libraries as an engine of social transformation and a space for creating communities that foster processes of open participatory research (citizen science) and solve local challenges. At the same time, it aims at empowering librarians with tools and methodologies needed for facing the change. One of the challenges identified was “Quality of air”: the idea is to get citizens closer to the current reality of air pollution, within the wider framework of climate emergency. The objective is to adapt public libraries to cultural and social changes that arise from the digital society, favouring the creation of collaborative and participatory environments open to citizens
11	Canòdrom Obert	City Council of Barcelona—Dept. of democratic innovation and Collectic Cooperative (technical bureau)	Canòdrom Obert is a set of open activities proposed within the framework of the venue Canòdrom—Digital and Democratic Innovation Centre. Canòdrom Obert allows to work in a closer way with the surrounding environment and the wider city. It integrates the cultural programming and the projects of the Centre, the activities in collaboration with the neighbourhood and the resident projects: exhibitions, workshops, research seminars, conferences, meetings, etc. that arise from the same ecosystem of the project and the collaboration with other actors. Canòdrom Obert is designed and articulated in a co-participated way by the associative networks and the neighbour community, the research residencies, and projects at the Digital and Democratic Innovation Centre and the AIDD Technical Bureau
12	Estem Rodant	Nadir Association	“Estem rodant” is a project aiming at working cinema in secondary schools and producing a collaborative film among the centres of different districts of the city of Barcelona. The project has been carried out in three different secondary schools of the city, located in different districts: Escola l’Esperança (Baró de Viver), Institut Lluís Vives (Sants) and Institut Salvador Espriu (El Clot). Each project has been carried out in different trimesters. “Estem rodant” is a project promoted by Nadir, an association made by four filmmakers, that carries out an educational project on the audio-visual medium with children and young people. Nadir organises workshops in collaboration with educational centres, leisure spaces or social entities, founding its action on practical and experiential work with all the participants. Nadir is also specialised in the documentation of educational projects and artistic processes that require an audio-visual record and language
13	Ciutat Esperança	elParlante Cultural Association	How do we see our neighbourhood and how do we want to showcase it? This has been the core question of Ciutat Esperança, an edu-communication project based on the use of mass and alternative media, that since 2013 aims at promoting critical thinking, information, expression and debate, sensitising on the complexity of different realities, mainly in the Ciutat Meridiana neighbourhood, in the Nou Barris district of Barcelona. In 2013, the Ciutat Meridiana Community Plan approached elParlante association with the aim to commission the dynamization of a group of youngsters in the neighbourhood. elParlante started a participatory reflection around imaginaries built by these youngsters, by critically revising with them the discourses of media and from there to elaborate new stories. The project is designed and managed by elParlante Cultural Association and since its start has the main aim to enhance the good things occurring daily in the territory and to give value to social and cultural diversity
14	MODUL Cultural and Ecological Centre	Contorno Urbano Foundation	The MODUL cultural & ecological centre was built in 2020 to make the Park a more resilient space that responds to the challenges and needs of the territory. This space creates new opportunities in the territory, giving a physical space for the neighbours to promote innovative community processes and create new work networks for community well-being and access to culture, education, or leisure MODUL is a flexible and innovative centre that works as a multidisciplinary venue. It has been designed with the project’s monitoring commission and began to host educational, leisure and cultural activities from November 2020

After the selection process, the authors contacted the different directors and project managers responsible for each organisation and sent to them, first, an online form aimed at collecting basic information on the project and its social impacts, followed by in-depth semi-structured interviews which allowed to further explore the researched topics. The interviews have been then examined through thematic analysis of qualitative data, being a suitable methodology to search for common or shared meanings (Kiger, Varpio 2020). The collection and analysis of cases have been carried out from October 2020 until November 2021.

Meanwhile, two workshops with local stakeholders have been organised in May and July 2021. The first event took place within the framework of the FAACCC Festival of Community Arts, organised by Basket Beat association, with the aim to identify the enabling factors of social transformation. The debate explored in three different round tables relevant themes such as key resources, the creation of synergies with other entities and projects, and the role of contextual factors and the empowerment of marginalised collectives.

The second event was a closed-door session on the topic of the assessment of social impacts of cultural projects, that saw the participation of the Culture Institute of Barcelona (ICUB); private foundations funding cultural projects aiming at social transformation (Art for Change Programme of La Caixa Bank) and directors of local cultural projects with social impacts. The discussion tried to merge the different needs of assessment regarding the social impacts of cultural initiatives in order to explore the typologies of indicators that best suit this purpose.

The reflections emerging from the above-mentioned process helped nurture the analysis of the Barcelona environment, through the identification of endogenous and exogenous factors affecting the generation of social impact in the analysed cultural projects, through the application of a logic model. The model has been adapted from the ‘Program Action—Logic Model’ developed by the Wayne State University (2014) and allowed to visualise for each project: the inputs (i.e. human, material and economic resources, governance model and values), the activities (including a classification of typologies of programme, organisational strategies, and the target groups involved), the outputs (i.e. the main qualitative and quantitative results of each project), the expected outcomes (categorised according to the three impact dimensions of MESOC: health and wellbeing, civic engagement and participation, and urban and territorial renovation), and the external factors (namely, conditions that influence programme success, such as the existence of explicit public and private policies, the socioeconomic scenario, and the composition, social values and dynamics of the cultural ecosystem). The relationships between the different components have been then examined through the lens of a theory of change approach (Anderson 2005), which helped to identify possible pathways of change.

## Exogenous factors

The development of determined cultural initiatives depends on the context these operate in. The main exogenous factors affecting cultural projects with a social orientation emerging from our research were the incidence of (a) the urban and socioeconomic context; (b) social values and dynamics of the cultural ecosystem; (c) public policies; and (d) supporting policies from philanthropic institutions.

### Incidence of the urban and socioeconomic context

The urban fabric, the social, demographic, and economic structure, as well as the emerging dynamics, have a high importance in the rise and evolution of cultural projects aimed at social transformation. The arrival of two big migratory waves coming from outside the European Union—the largest one during the decade prior to the 2008 crisis and the second one from 2014 until the global pandemic—represents the main demographic and social change of the Barcelona metropolis (Domingo et al. 2021). New migrants settled in specific spaces of the urban fabric of Barcelona, mainly in deprived areas of the city centre and in the outskirts of the metropolis. The economic crisis hit a large part of the over one million new residents particularly hard, a fact that explains the rise of several social and cultural initiatives to mitigate its negative effects.

The case of Ciutat Meridiana well exemplifies how the confluence of the economic crisis, migrant waves, socioeconomic deprivation, and urban planning may affect a given territory. The small neighbourhood is the Northern gate to the city of Barcelona and represents the failure of an urban model based on property speculation, which led the most vulnerable neighbours to occupy apartments left empty by the economic crisis, and now owned by banks. The housing issue has earned Ciutat Meridiana the title of ‘Eviction City’. It is in this context that the Ciutat Esperança project was born, with the specific aim to defy the current negative narratives of the neighbourhood, from the hands of the local youngsters and with the help of the audio-visual medium.

Some of the projects analysed in our research work with collectives affected by the abovementioned dynamics, both at the territorial level—acting in multicultural neighbourhoods such as, for instance, El Raval, Santa Coloma de Gramenet, or La Florida in the case of Xamfrà, Cuines del Món, and Contorno Urbano projects, respectively—and in terms of origin of migratory flows (Latin American refugees and migrants living in Barcelona, in the case of En Palabras). In some cases, the urgency to dedicate the project specifically to disadvantaged communities may lead to relocating the organisation in terms of space. This is the case of L’Arc Music Foundation (the organisation that develops the Xamfrà project), which moved part of its activities from the upper-class neighbourhood of Sarrià to the multicultural Raval and other working-class neighbourhoods.

Gentrification, a generalised phenomenon in big capitalist metropolises, is linked as well to several cultural dynamics (Yúdice 2008). In the analysed context, this came along with the big touristic flows (before the Covid-19 pandemic, Barcelona was the fifth most visited European city for number of international tourists^3^) but has been associated also with urban regeneration operations connected with the concentration of cultural venues or the creation of creative spaces, as in the case of the Raval neighbourhood and the 22@ District. Despite the opposition of certain citizen collectives and some public interventions to mitigate the negative effects of the phenomenon, the result has been ambivalent: the displacement of local residents occurred, and, at the same time, a mix of economic and bohemian migrants have been attracted (Quaglieri-Domínguez, Russo 2010). The dialectic relationship between residents, new residents and non-residents and the dynamics of gentrification have been the core topic of the community creation led by Art i Part programme with the neighbours of Poble Sec. The residents worked on the themes of loss of identity and urban transformation, but also the richness of diversity, in a number of workshops that culminated in a public performance.

### Social values and dynamics of the cultural ecosystem

The Catalan society of the twenty-first century is characterised by increasingly individualist values and, at the same time, the emergence of alternative collaborative movements with social vocation (Elzo, Castiñera 2012). Cultural creation swings between these two values, being sometimes very autoreferential and other times at the forefront in terms of social commitment. The analysed projects could be considered part of this second group. Indeed, the creation of the FAACCC—Community Arts Festival is a clear example of collective self-governance that, despite receiving public support, stands as a demanding and independent profile with respect to public powers. The festival is thus the result of a collective action of five groups of stakeholders: cultural and social entities, universities, neighbours, non-institutional collectives, and professionals.

This critical approach to cooperation between organised civil society and the administration characterises the ‘Barcelona model’, where a fabric of small alternative initiatives is complemented by the projects stemming from big cultural institutions (Rius-Ulldemolins, Klein 2022). Everyone knows each other and, despite the critical component, there exists considerable complementarity, even some degree of recognition and permeability, between cultural activism and institutional professionals.

On the other hand, the cultural sector is at the same time recipient and amplifier of predominant and emerging social values. A clear illustration of these tendencies could be found in the Womart and Estem Rodant projects, both embracing a gender perspective in the visual arts and audio-visual field respectively. Similarly, the proposals born within the framework of El Canòdrom incubator are built upon emerging values and trends, such as open technologies and digital rights, feminisms, community participation and revitalisation, and digital culture and videogames. The attention to ecological issues and the climate crisis is also evident, for instance, in the ISC2 project, which revolves around the topics of air pollution and climate emergency to foster processes of open participatory citizen science. In this sense, public libraries reposition themselves as agents of cultural and social change for the local communities, functioning as gateways to knowledge and inclusion and as central cultural and public spaces (European Parliament 2016).

The ability to connect in networks with other similar movements at the international level is also noteworthy. Indeed, the presence of a large community of foreign students and young professionals, particularly from Europe and Latin America, reinforces relationship networks, mutual influences and connectivity with similar initiatives developed in other parts of the world.

Finally, it should be taken into account the existence of a strong social and political consensus around the social value of culture. According to a survey of three socio-economic typologies of the city's neighbourhoods (Barcelona City Council 2020), citizens shared positive perceptions on this matter, despite slightly differing assessments of what culture means to each of them. Another clear example of this can be found in the Declaration of the Catalonia Government proclaiming culture as an essential good (September 2021),^4^ a fact that has allowed to keep cultural activities open during the restriction periods due to the pandemic. In parallel, the Barcelona municipality also embraces the commons as a key paradigm for social and cultural change, adopting the ‘City as a Commons’ lemma (Foster, Iaione 2016) that enables plural and diverse perspectives to emerge. Canòdrom Obert embodies this political strategy, especially through its digital rights strand of activities, by providing an open space for participatory democracy laboratories and debate.

### Incidence of public policies

The results of the 2015 municipal elections granted the victory to *Barcelona en comú*, a left-wing confluence led by the new mayor—the social activist Ada Colau—who promoted several initiatives focused on favouring a more direct citizen participation. However, the government agreement with the socialist party failed to substantially transform the inertia of the municipal public action (Barbieri 2018; Zamorano 2018). In the area of cultural policies, after a first impetus, new ideas are hardly translated into a change of priorities in the municipal budget (Rius-Ulldemolins, Gisbert 2018).

The election of Joan Subirats as new councillor for education and culture during the second mandate in 2019 reinforced the relevance of the social dimension of cultural policy. Since then, the city government has demonstrated a strong commitment with the general topic of social impacts of culture, and in particular with citizenship and cultural rights. In terms of cultural policies, this is reflected in the most recent cultural plan named ‘Fem Cultura’, officially presented on April 30, 2021, that aims at designing public policies from the perspective of cultural rights and opening the path towards the recognition of these rights for all citizens of Barcelona. The plan presents 9 measures and 100 actions to be carried out until 2023, and give special emphasis to the topics of interculturality, feminism, transparency, transversality, decentralisation and new centralities, metropolitan culture, and sustainability^5^.

This political strategy has impacted on the criteria for grants’ provision, prioritising those projects focusing on cultural diversity and fostering active citizen participation. This bottom-up perspective, more centred on the cultural democracy paradigm, although does not question the amount of budget allocated for cultural democratisation, has allowed to prioritise projects with an explicit social impact. Also, support has been given indirectly to peer-centred initiatives through the use of public premises—as in the case of Xamfrà—or the reorientation of the programming strategies of cultural venues and events—in the case, for instance, of Apropa Cultura.

### Incidence of support policies from philanthropic institutions

Amongst philanthropic institutions, private foundations play a prominent role in terms both of external support to cultural projects with strong social impact and promotion of own projects with the same orientation, as it is the case for Explainers and Primer Cinema projects. Two of the most relevant cultural foundations based in Catalonia, La Caixa Foundation and Carulla Foundation, have developed in the last years specific lines in this direction. Their work consists not only in providing economic contributions to the highest valued initiatives through their respective support programmes, but also in ensuring visibility to the projects and offering spaces for education and shared reflection to their managers. This role of accompaniment is important to strengthen a still emerging sector in the Catalan cultural panorama, and at the same time, it aligns and reinforces the municipal strategies to foster cultural rights to all citizens. This orientation, born as an answer to the effects of the 2008 financial crisis, is reinforced by the crisis provoked by the Covid-19 pandemic. Culture is not only perceived as an essential activity for human beings, but also as a fundamental means for citizen health and wellbeing. In this sense, other philanthropic institutions—more centred on educational or even alimentary strategies, like the Daniel Foundation and Nina Carasso Foundation—are fostering community art projects.

Table 2 synthesises the main exogenous factors detected from the case analysis, that operate as drivers to boost the development of cultural projects aimed at generating social impact. As it can be observed in the numbering^6^ included in the table for each factor, all the projects analysed in the Barcelona case are in part the result of the existence of these preconditions. Nevertheless, whilst many factors (like the existence of a collaborative environment or the recognition of successful cultural projects aiming at social transformation) have an impact on most projects, as a result of the interdependent and mature Barcelona ecosystem, other factors only affect specific projects. This is probably due to the specificity and mission of each institution and project involved, which represent an endogenous aspect to take into account and that dialogue interdependently with the social and political dynamics of a given territorial context.

**Table 2:** Exogenous factors

Exogenous factors		Evidence from cases
Urban and socioeconomic context	• Urban structure and connectivity • Existence of socio-economic disparities in the area • Multicultural environment • Gentrification	• 3,8,9,10,13,14 • 1,2,3,4,7,8,9,11,13,14 • 2,3,4,7,8,9,11,13,14 • 2,3,4,7,9,12
Social values and dynamics of the cultural ecosystem	• Recognition of successful cultural projects aiming at social transformation • Commons as a social strategy • Sensitivity towards equity at societal and cultural professional level • Cultural activism • Collaborative environment	• All projects • 1,2,3,4,7,8,9,10,11,13,14 • all projects • 2,3,4,7,8,11,13,14 • All projects
Incidence of public policies	• Existence of specific budget allocations to support cultural projects with social impact • High interdepartmental collaboration • Co-participation of diverse organisations and institutions in the development of public policies • Strong discursive and political leadership for a social agenda in culture	• 1,2,3,4,7,8,9,10,11,12,13,14 • 1,2,3,4,8,9,11,13,14 • 1,2,3,8,11,13,14 • 1,2,3,4,6,7,8,9,10,11,12,13,14
Supporting policies from philanthropic institutions	• Existence of specific budget allocations to support cultural projects with social impact • Mentoring and training accompaniment • Discursive and lobby commitment merging social and cultural dynamics	• 1,2,3,4,5,6,8,14 • 5,6,8 • 2,3,4,5,6,8,14

## Endogenous factors

Beyond the contextual conditions, several endogenous factors explain and facilitate the development of cultural projects with social impact. These conditions are strictly related with the strategy and beneficiaries of each project. Our research identifies three large categories of endogenous factors: (a) governance model and organisational culture; (b) human resources, its values and management; and (c) symbolic, material and financial resources.

### Governance model and organisational culture

Most of the analysed cases are carried out by independent cultural entities, either associations or, in some cases, small foundations. This fact is the result of the rich social and cultural Catalan fabric, in tension and at the same time in collaboration with the public administration and some big cultural foundations.

The nonconformist and activist character and the desire for autonomy of the social actors do not remove the dependency from the public administration in terms of material and financial resources. But the organisational culture and values are horizontal. This fact is evidenced in the internal organisation of projects and entities, with participatory leadership models. A remarkable case in this sense is Basket Beat, whose governance model is based on transparency among all participants, including interns and volunteers.

On the other hand, alongside the independent sector, the public administration is also committed with projects of direct cultural management. Amongst the analysed projects, this would be the case of the ones carried out by public libraries or municipal cultural venues. For instance, the libraries’ consortium of the Barcelona Province is responsible, together with the City Council of Santa Coloma de Gramenet, for the Cuines del Món project. Within the Biblio-Lab framework of the Barcelona Province as well, ISC2 is a networking project implemented by three metropolitan libraries. In this case, it is noteworthy to highlight the advantages and synergies derived from sharing resources and expertise resulting from the fact of being part of a network of venues.

In all those cases, the public professionals behind each initiative play a very important role. A good example of this is the Apropa Cultura project, an initiative of one of the professionals of the Barcelona Auditorium that, with the initial support of the director, crossed the threshold of the Auditorium. It currently involves 125 public and private cultural programmers in 53 Catalan municipalities, receiving people in a vulnerable situation proceeding from more than 2.300 social centres. This case is certainly the project with wider social impact, in terms of number of beneficiaries over the last 15 years, probably also because it is aligned with the mission of the majority of cultural venues, under the paradigm of cultural democratisation. However, without a horizontal management model, it would have not found consistency with the entire public system, that bet on scaling up a successful initiative.

More related to a partisan strategy is the case of the Canòdrom Obert project, which forms part of the municipal Digital and Democratic Innovation Centre, an initiative of the current Barcelona government. The proposal is in line with the new political priorities and the participatory model and governance of the current municipal government, but it also falls back on many grassroot cultural actors, fostering a quadruple helix approach that involves universities, administration, organisations, and civil society.

Finally, we have the case of cultural projects developed by La Caixa Bank Foundation. Primer Cinema and Explainers are examples of projects directly developed by the foundation, and not related to the support programmes addressed to independent projects (like ‘Art for Change’). The insertion in its own programme of activities allows not only to provide sufficient resources to move the projects forward, but also that the experimentation brought to the organisation probably explains a change in the priorities of the support programmes to external projects.

### Human resources, its values and management

All interviewed directors remarked the importance of the human team as a success factor of the projects, above the availability of financial or material resources. With limited material resources it is still possible to achieve great results, but without motivated and committed professionals, this would not be feasible. Personal commitment and values, along with the cumulative expertise of professionals and the networking capacity are fundamental. ‘Having strong ethical factors is fundamental’ states Ester Bonal, director of Xamfrà, since ‘ethics is like the funambulist rope: an intangible but powerful resource’. She is not the only one to defend this position since other projects’ directors advocate for a culture of equity.

In addition to these factors, relying on a diversity of people in terms of staff composition is key to generate fruitful exchanges and empathy, as in the case of Basket Beat, that expressly aims at generating a culture of equity and diversity in the team by considering gender and ethnic criteria in the recruiting process. Also noteworthy is the freedom given to interns and participants for learning and proposing how they can help the project. Two of the analysed cases—Contorno Urbano and Explainers—heavily rely on volunteers. This widespread strategy in the cultural sector is considered one of the most effective for increasing the civic commitment of citizens, despite having different degrees of implication. The same concept of cultural volunteering has different nuances, ranging from strong commitments to more informal and spontaneous participation (Calvano 2021).

### Symbolic, material and financial resources

Another key factor is represented by the availability of adequate symbolic, material and financial resources to fulfil the ambitions of each project.

The existence of powerful symbolic resources (for example, the recognition given by an award, developing activities in an emblematic building, or having an opinion leader within the organisation) has positive effects both in terms of good reception of the project by the community and beneficiaries, and capacity to attract other resources.

Undoubtedly, the scarce prioritisation (maybe related to a limited visibility and quantitatively reduced impacts) explains the reduced budgetary dimension of the analysed projects. Raising funds from different institutions ends up being a relevant asset, even in large-scale projects such as Apropa Cultura, which has received wide recognition.

Some projects stress the importance of having an own (or ceded) venue that gives them visibility and allows them to position as benchmarks in the neighbourhood. For instance, Xamfrà explained how their work changed since the municipality ceded them a space in the centre of the Raval neighbourhood (‘a door open to everyone’). This openness to the community is considered crucial also for the MODUL project, that gives access to their premises to all entities and collectives in the neighbourhood willing to take part in the project, by literally providing them with a set of keys.

In other cases, organisations opt for using material resources and premises of collaborating institutions (e.g., educational, social, or cultural centres), that allow to save costs and focus the efforts, generating networks of entities and professionals that multiply the impact of their work.

In unique projects, such as Cuines del Món, having specific material resources—in this case, a kitchen located inside the library—constitutes the enabling factor for proposing a different project and programming. In this case, the traditional culture—books—is linked with the popular culture—gastronomy. This material asset changes the dynamics of a library located in a multicultural and working-class city like Santa Coloma de Gramenet. Other projects, like Basket Beat, decided to work with a very accessible and economic resource—a basketball—and use the synergy between sport and culture as an enabler to reach non-conventional participants.

Table 3 summarises the main endogenous factors emerged from the analysis of the cases in the Barcelona ecosystem^7^. As for the exogenous factors (Table 2), there are some conditions that affect the totality of the cases studied—for instance, the previous experience of the organisation in cultural projects with social impact or the strong commitment of the team towards social impact capacity—whilst other have been highlighted only in a given number of projects (e.g., the presence of an open and participatory governance model). As previously stated, this may be due to the specific characteristics and mission of each project, impacting on the organisational strategy.

**Table 3:** Endogenous factors

Endogenous factors		Evidence from cases
Governance model and organisational culture	• Open and participatory governance model • Previous experience of the organisation in cultural projects with social impact • Networking and synergic mindset	• 2,3,8,11,13,14 • All projects • All projects
Human resources	• Personnel trained and/or with expertise in social impact projects • Capacity to attract and retain a committed, motivated and talented human team • Strong sensitivity/commitment of the team towards social impact capacity • High diversity of profiles in team composition	• All projects • 1,3,5,13,14 • All projects • 2,3,8,11,12,14
Symbolic, material and financial resources	• Legitimation of social impact through institutional prestige, media coverage and pervasiveness • Capacity to access adequate funding • Access to a diversity of financing sources • Availability of an own or ceded venue and material resources	• 1,2,3,4,5,6,8,14 • 1,5,6,8,9,10,11 • 1,2,3,4,8,13,14 • All projects

## Discussion

**Fig. 1:**
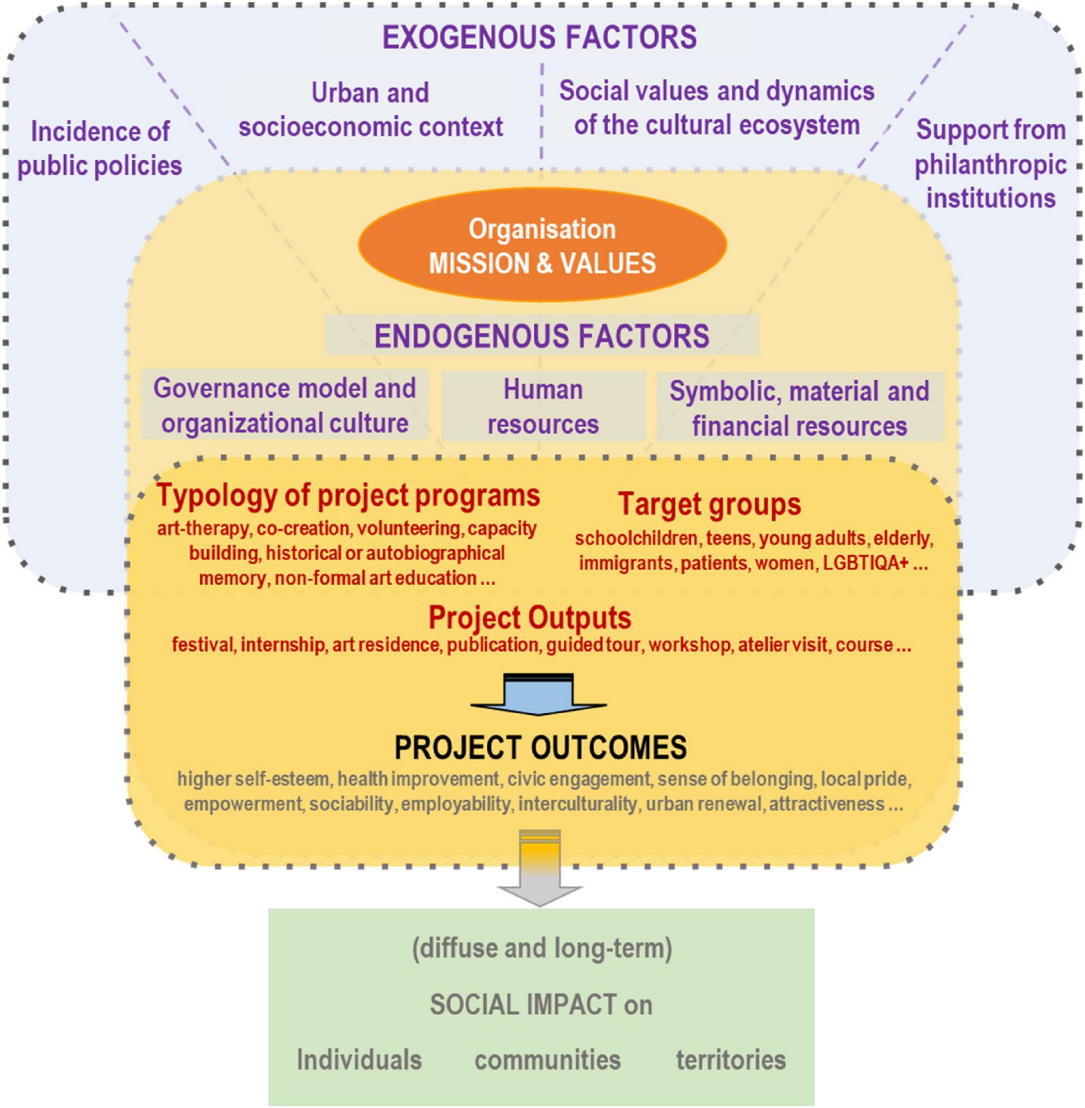
Exogenous and endogenous factors affecting the social outcomes and impacts of cultural projects

Both endogenous and exogenous factors condition and are strictly related to the mission, values and strategy of each organisation and project. This fact influences the type of programmes selected and the target groups involved by each institution behind the analysed projects, as well as the outputs and outcomes obtained. Thus, it is useful to identify the typology of cultural programmes and the most common beneficiaries in order to highlight common points and singularities. At the same time, it is important to bear in mind that the social impact on individuals, communities and territories is always long-term and a result of a multiplicity of factors that escape what a single project can achieve. For this reason, our analysis focuses on the influence of specific exogenous and endogenous factors, as well as the selection of programmes, target groups and project outputs, on the outcomes obtained (Fig. 1).

Regarding the typology of cultural programmes, many of these strategies are used simultaneously since synergies are generated through combination. Many projects combine strategies and methodologies developed in other contexts or sectors (for instance, participatory strategies or capacity building). In other cases, the projects create their own methodology, developed starting from the observation of the mediation practice, as in the case of Basket Beat (Aragay, Sitges 2020). In any case, strategies are key to achieve the expected outcomes, and thus advancing in the social impact that justifies their existence.

One of the most common intervention strategies among the observed cases is centred on non-formal education and artistic capacity building, in line with what is occurring in many Western countries (Jones, Perry 2019, Matarasso 2019). The analysis also detected a great variety of experiments related to community co-creation and co-acting (for instance, the case of Art i Part for the performing arts sector), the co-design or co-curatorship and co-management or co-production of events, being the first one the most common option. This second group is part of a tradition started by community arts back in the 1970s (Matarasso 2019), but that became widespread during the second decade of the twenty-first century, boosted by social, political, and technological streams (Bonet, Négrier 2018b).

Said so, to what extent the selection of a specific strategy depends on the typology of users this is addressed to? Surely, in the case of child users, it is clear that the strategy should be adapted to their comprehension abilities and their capability to interact with different age groups. Similar considerations should be taken into account in the case of groups of people with specific illnesses or cognitive or physical impairments, who represent collectives usually involved in art-therapy projects aimed at increasing wellbeing or improving health through cultural activities.

Nevertheless, projects that could be based on standardised methodologies prefer to select a specific strategy for working with their communities of reference. It is the case of En Palabras, that expressly chooses the writing of autobiographical stories as the most adequate means to work with migrant collectives and provide them a visibility space. In contrast, elParlante association seems to hint that the methodology (and the cumulative experience of professionals and organisations) is easily applicable to different collectives.

Most of the results of the analysed projects (with the notable exception of Apropa Cultura) end up affecting a relatively small number of people, their communities and territories. However, the effect can be profound in aspects such as higher self-esteem, health improvement, civic engagement, sense of belonging, local pride, citizen empowerment, sociability, employability, interculturality, urban renewal or local attractiveness, among others.

Another relevant aspect emerging from the studied cases is the sustainability, the synergies and the feedback between policies, cultural projects, and social projects. An example of the latter is found in projects such as Art i Part or Apropa Cultura, that are based on the interaction between the cultural and social fabric to achieve the desired social impact. Additionally, the capacity to influence public policy is a key factor. In this sense, the critics from the professional sector addressed to the Art i Part municipal initiative, that eventually disappeared, or the capacity to organise the FAACCC—Community Arts Festival are examples of the coordination capacity of the sector and the difficult but fruitful relationship between an organised civil society and the public administration, that characterises the ‘Barcelona model’ (Rius-Ulldemolins, Klein 2022).

Finally, it is worth noting that the synergic potential of some of the analysed projects produced unexpected spillover effects such as the creation of replicas or the decision of the addressed collectives to start new initiatives thanks to the experience obtained in the project. The latter is the case of Meri Productions, an audio-visual production association born from the initiative of the youngsters who took part to the Ciutat Esperança project, or the case of En Palabras, where ex participants to the literary workshops created an informal collective in order to maintaining their relationship with the project through the development of parallel activities. The synergic potential is an element that appears throughout the different analysed cases at different levels, from the interactions with universities (the university Nutrition Centre in the case of Cuines del Món) to the connections with other territories (for instance, the Balearic Islands in the case of Apropa Cultura).

## Conclusions

Most of the exogenous factors analysed in this research present a similar impact capacity (although with varying degrees) in all the analysed projects, making it hard to determine whether it came first the existence of a political strategy and of projects in line with it but independent in nature; or the existence of shared values and a social fabric that are at the base of the emergence of supporting policies and cultural initiatives with social impact. In this sense, the analysis carried out shows that it is not possible to separate the growing number of this typology of projects emerged after the 2008 crisis and the appearance of public—as well as philanthropic—policies without taking into account the specific social, cultural, economic and political local context. The pre-existence of experienced professionals and cultural institutions sensitive to social issues is the ideal breeding ground for the emergence of these new projects and supporting policies.

Another question is if we should talk about a strictly local phenomenon and model, born in the shadow of the 2008 crisis and intertwined with the Barcelona social fabric—an updated replica of the tradition of tense cooperation between civic initiatives and the public sector that characterises the 'Barcelona model'-, or if it could be generalised. This question is difficult to solve through case studies since it would involve making comparisons with other contexts. Our hypothesis, in the light of the Barcelona case, is that exogenous factors condition the emergence of new projects and support policies. But, at the same time, there exist influences that easily cross borders, as it is demonstrated by the generalisation and evolution of the big paradigms and strategies of cultural policies in Western democratic countries (Hadley, Belfiore 2018, Bonet, Négrier 2018a). The growing importance of factors such as social or economic impacts in cultural policies can be seen as an instrumentalisation by other public policies. Nevertheless, the social impact (more than the reductionist economic perspective) is perceived by certain cultural sectors as a positive value of commitment of cultural institutions towards society.

We may also question the relationship between some of the endogenous factors with the territorial context. If the human capital is widely considered by the analysed projects’ leaders as a fundamental factor, differences may be appreciated in terms of the availability and use of material and financial resources. In this sense, the different perceptions around the fact of having an own space that make visible and facilitate the territorial insertion of the project is noteworthy. Indeed, although all analysed projects disposed of own or ceded venues, this was not considered as relevant as having a committed human team. Conversely, the ownership of the project does not seem to be a differentiating factor, since we found both projects directly managed by the public sector or philanthropic foundations with considerable resources, along with independent grassroot projects, with less resources and poor stability. Nevertheless, a collaborative organisational culture and values and strong commitment of the human team towards social causes represent a common denominator in the examined cases.

As a final remark, the considerations regarding endogenous and exogenous factors presented in the article may be of wider scientific and political interest. In the first case, the research in other geographical contexts will be useful to corroborate the validity of the enabling conditions identified for studying social impact generation in the case of cultural projects. In the latter, the analysis of the factors could be accompanied by the construction of context-specific indicators, which may orient local cultural policies and the granting of subsidies addressed specifically at cultural projects aiming at social transformation.

## Data Availability

The datasets used and/or analysed during the current study are available from the corresponding author on reasonable request.

## Footnotes

1 Art i Barri is the Catalan network of professionals, entities and collectives committed to art-making as a means to improve the lives of people and communities.

2 The temporal delimitation is justified by the importance of Spain's municipal elections of May 2015, when a constellation of new political forces emerged all over the country. ‘For the first time in almost 40 years of Spanish democracy, the country’s major cities would no longer be ruled by either the Partido Popular (PP) or the Partido Socialista Obrero Español (PSOE), or any of the other long established political forces, but by new ‘Municipalist Confluences’ such as Ahora Madrid, Barcelona en Comú, and Cadiz Si Se Puede, to name just a few’ (Rubio-Pueyo 2017: 2). In Barcelona, the rise of Barcelona en Comú marked a turn in the political orientation of the City Council, with a strategy for promoting social rights.

3 Data retrieved from Barcelona Activa (2014).

4 https://govern.cat/salapremsa/notes-premsa/387950/el-govern-declara-la-cultura-be-essencial.

5 https://www.barcelona.cat/barcelonacultura/ca/recomanem/fem-cultura-pla-drets-culturals.

6 The numbers included in Table 2 refer to the 14 analysed projects of this article. The numbering follows the order of Table 1.

7 As for Table 2, the numbers included in Table 3 refer to the 14 analysed projects of this article. The numbering follows the order of Table 1.
